# Physiotherapy according to the BeBo Concept as prophylaxis and treatment of urinary incontinence in women after natural childbirth

**DOI:** 10.1038/s41598-021-96550-x

**Published:** 2021-09-10

**Authors:** Aneta Śnieżek, Dorota Czechowska, Marta Curyło, Jacek Głodzik, Paweł Szymanowski, Anna Rojek, Anna Marchewka

**Affiliations:** 1 Department of Clinical Rehabilitation, University of Physical Education in Krakow, Krakow, Poland; 2 Department of Physical Medicine and Biological Recovery, University of Physical Education in Krakow, Krakow, Poland; 3 Department of Gynecology and Obstetrics, Andrzej Frycz Modrzewski Krakow University, Krakow, Poland; 4 Gabinet Fizjoterapii ReSport, Tarnów, Poland

**Keywords:** Rehabilitation, Medical research

## Abstract

Pelvic floor muscle dysfunctions can lead to urinary incontinence, a condition which often affects women both during pregnancy and after childbirth. As a result of this, certain exercises are recommended during and after pregnancy to prevent and treat this incontinence, and the BeBo Concept is one of these methods used to prevent pelvic floor muscle dysfunction. The aim of the present study was to evaluate the effects of a 6-week course of physical therapy according to the BeBo Concept on the improvement of perineal muscle strength and endurance as well as urinary continence in women after their first vaginal delivery. The study was conducted on a group of 56 women who were randomly assigned to the exercise (n = 30) or control (n = 26) group. The exercising group participated in a 6-week physical therapy program according to the BeBo Concept. Pelvic floor muscles were assessed using the perineometer and palpation Perfect Test. UDI6 and ICIQ-SF questionnaires were used to obtain information about the symptoms of urinary incontinence, evaluate the frequency, severity and impact of urine leakage on the quality of life. In all women after natural childbirth, regardless of treatment, it was observed that measured parameters improved, but the improvement was slightly more explicit in those who participated in the Bebo Concept exercise group (e.g. ICIQ-SF exercise group *p* = 0.001, control group *p* = 0.035). Due to its positive impact on the pelvic floor, this exercise program should be recommended to women after natural childbirth.

## Introduction

Urinary incontinence among women is considered a social disease, which may affect up to 20–60% of the population of women over 18 years of age^1^. In 2010, The International Continence Society and The International Urogynecological Association defined urinary incontinence as a “symptom”, a complaint of the involuntary loss of urine^2^. Its most common form in women is stress urinary incontinence^3^. It is characterized by the involuntary loss of urine during physical exertion, eg sports activity, or when coughing or sneezing^2^. Natural childbirth, especially the first one, severely affects the anatomy and functioning of the pelvic floor muscles (PFM). Maternal characteristics at childbirth, such as age and high BMI, increase the risk of PFM dysfunction, but birth factors also play an important role. In women with severe perineal trauma, symptoms of PFM dysfunction are reported shortly after delivery^4^. Mechanical injury to the perineal muscles is sometimes irreversible. Research shows that within 8 months of delivery, some women did not regain their PFM strength, and 34% of them were unable to voluntarily contract the perineal muscles 6 weeks postpartum^5^. Jundt et al. confirmed that the bladder neck in women with postpartum stress urinary incontinence was much more mobile than in women who were able to hold their urine^6^.

The strength and endurance of the pelvic floor muscles decrease significantly after the first delivery^7^. Research shows that 65% of women with urinary incontinence remember that the first episode of urine loss occurred during pregnancy or in the puerperium^8^.

The researchers noted that after natural childbirth, more than one-third of asymptomatic women developed urinary incontinence, and this lasted one year after delivery in two-thirds of that group^4^. Other reports show that 3 months after childbirth, about one-third of women experience urinary incontinence^9,10^. Subsequent observations provide general data that 1 in 3 women reports urinary incontinence after delivery^11,12^, even up to a year later^4^. Loss of urine during pregnancy is the main risk factor for the occurrence of stress urinary incontinence after the first vaginal delivery^12–15^.

Postpartum remission of stress urinary incontinence was also observed and this can be explained by the resolution of hormonal and metabolic changes related to pregnancy and spontaneous healing of traumatic injuries resulting from natural delivery^13^.

The use of postnatal prophylaxis plays an important role in restoring the function of the pelvic floor muscles as well as preventing pelvic organ prolapse. That is why education among women concerning the perineal muscle is so important.

The BeBo pelvic floor training, in the original: BeBo Gesundheitstraining, was established in Switzerland and is conducted in the form of group workshops. The method is currently one of the concepts that is used to prevent pelvic floor muscle dysfunction. It is also used in treatment of urological and gynecological disorders in women and men. The advantage of this method is a holistic approach to the human body, paying attention not only to the activation of the pelvic floor muscles, but also to correct body posture, which in turn affects the functioning of the perineal muscles^16^. At the beginning of the workshop each participant is asked to fill in health questionnaire, including information on clinical conditions, urinary incontinence symptoms, and others.

The idea of the method is based on five basic groups of exercises: awareness, mobilization, strengthening, relaxation exercises, and the integration of pelvic floor muscles into everyday activities^16,17^.

The aim of the present research was to evaluate the effects of 6 weeks of physical therapy using the BeBo Concept on the improvement of perineal muscle strength and endurance as well as urinary continence in women 6 to 8 weeks after the first vaginal delivery.

## Methods

The research was approved by the Bioethics Committee at the Regional Medical Chamber in Kraków (No. 151/KBL/OIL/2017 of 22/09/2017). Prior to the study, all participants were informed of the purpose and method of conducting the research. Each of them signed an informed consent release to participate in the project and to process personal data for scientific purposes. All procedures were perfomed in accordance with the Declaration of Helsinki. We confirm that all research was performer in accordance with relevant guidelines and regulations.

### Eligibility/qualification

The research was carried out among a group of 56 women 6 to 8 weeks after their first vaginal deliveries. Patients were recruited from three Krakow hospitals, from obstetrics departments.

The criteria for inclusion in the project were as follows: primiparous females with a single pregnancy, 20–40 years old, delivery between 37 and 42 weeks of pregnancy, 6 to 8 weeks after natural childbirth, no contraindications to exercise stated by an obstetrician, voluntary participation in the study.

Exclusion criteria: multiple pregnancy, Caesarean delivery, postpartum complications in the form of: separation of the pubic symphysis and sacroiliac joints, thrombophlebitis, 3rd and 4th degree of perineal rupture, urinary tract or vaginal infections/infections during the experiment, 3rd and 4th degree of pelvic organ prolapse, 3rd degree of stress urinary incontinence or overactive bladder diagnosed before pregnancy, gynecological surgeries, spine operations, pelvic and spine fractures, injuries, operations on the lower limbs 12 months or less previous to the study, diseases of the nervous system, e.g. MS, stroke, respiratory diseases, diabetes, cancer, rheumatic diseases, or refusal to participate in the study.

Participants were randomized into two groups. They independently drew a card with the name of the group to which they would belong: control n = 26 and exercise (experimental) n = 30. Each participant, regardless of their group status, had a pelvic floor muscle assessment performed at the beginning and 6 weeks after the study.

### Digital palpation of PFM

Before the vaginal palpation examination of the pelvic floor muscles (PFM), all women were thoroughly informed about the procedure and gave their written consent.

Then, the participants were familiarized with the function of the PFM and taught how to properly tighten them. They were instructed to focus on the muscles being assessed, without involving the gluteal muscles, thigh adductors or external hip rotators. They were also asked to breathe freely^7^.

Subjective vaginal palpation to assess the function of the PFM^18^ with the PERFECT test was performed by a qualified physiotherapist. After washing and disinfecting the hands, the physiotherapist put on disposable gloves and applied gel to their index finger. One finger was inserted into the vagina along the length of two distal phalanges^19^.

The following items were verified: power, endurance, repetitions, and number of fast contractions of the PFM^20^.

### Perineometer test

The perineometer Peritron 9300 V (Laborie, Mississauga, Ontario, Canada) was used to objectively assess the functioning of the pelvic floor muscles^18^, and the following elements were analyzed: resting tension, maximum voluntary contraction and endurance of the pelvic floor muscles.

The test was performed in the supine position with the lower limbs bent at the hip and knee joints (up to 60°) and a pillow under the head.

A disposable condom was placed over the disinfected probe and the device was then inserted into the vagina. The center of the probe was placed 3.5 cm inside the vagina^21–23^. The rest value was recorded and the device was zeroed according to the instructions. Subsequently, the patients were asked to perform 3 voluntary maximal contractions of the pelvic floor muscles while holding the tension for 5 s. The intervals between contractions were 30 s^22–24^. The highest achieved value in cm/H_2_O was recorded for each contraction^25^. The mean of three trials was then analyzed^22^.

The next step was to assess the endurance of the pelvic floor muscles, in which the women performed a maximum contraction of the perineal muscles for as long as they could maintain it. The measurement was interrupted when the muscle tension decreased by 35% or more^20^.

### UDI-6 Questionnaire and ICIQ-UI Short Form

During the study the standardized questionnaires^26^ Urogenital Distress Inventory Short Form (UDI-6)^27^ and the International Consultation on Incontinence Questionnaire—Urinary Incontinence Short Form (ICIQ-UI) were used. UDI-6 was used to obtain information about the symptoms of urinary incontinence^27^ and to measure how troubling the symptoms are^28^. ICIQ-UI was used for evaluating the frequency, severity and impact of urine leakage on the quality of life^28^.

Both questionnaires were translated into Polish^28^. After 6 weeks, the patients completed the questionnaires again.

### Physiotherapy program using the BeBo Pelvic Floor Training Concept

Women from the exercising group participated in a 6-week physical therapy program according to the BeBo Pelvic Floor Training Concept. Individual meetings were held twice a week, each lasting 60 min. Each meeting consisted of a theoretical part lasting up to 10 min and a practical part lasting about 50 min. In addition, each participant received a set of exercises to be performed at home once daily.

In the first week (meetings 1 and 2), the homework included the following activities: concentration on the pelvic floor muscles and becoming aware of them in various body positions, mobilization of the pelvis in a sitting position on a chair—10x, lying on the back with bent legs and activation of PFM with exhalation, inhalation—relaxation—10x, balance exercise, standing on one leg, 10 s each leg.

In the second week of exercises (meetings 3 and 4), the previous homework was repeated and additional exercises were introduced: PFM activation up to 50% of the maximum strength and holding the tension for 3 s—5x, 5 s between repetitions, PFM activation up to 50% of the maximum strength and maintaining the tension for one breathing cycle—5x, the interval between successive repetitions was 5 s.

In the third week (5th and 6th appointments), daily home exercises consisted of repeating all previous tasks while extending the time of standing on one leg to 20 s.

In the fourth week of exercises (meetings 7 and 8), in addition to repeating the previous training, the following was introduced: activation of the PFM up to 50% of the maximum strength and maintenance of tension for 5 s—5x, interval between subsequent repetitions 5 s, activation of the PFM up to 50% of the maximum strength and maintaining tension for 2 breathing cycles—5x, several breaths between repetitions.

In the fifth and sixth weeks (meetings 9, 10, 11, and 12), the participants were asked to complete all the previous tasks, the time standing on one leg was extended to 30 s.

Attention was also paid to breathing exercises, balance exercises and learning the correct posture. The following aids were used: a large and small ball, balance disc, foam roller.

Exercises were conducted in various starting positions, incl. standing, kneeling, lying on the side, lying on the back, etc.

The control group did not participate in any research procedures between the baseline and the end of the study.

### Statistical analysis

All measurements were subject to statistical analysis carried out using the computer statistical package Statistica. In all calculations, *p* < 0.05 was considered statistically significant. The results of quantitative parameters were described taking into account mean value, standard deviation, median value as well as minimum and maximum value. Tests were also carried out for the compliance of the distribution of the parameters with normal distribution (Shapiro–Wilk test). Results for values resting pressure of the PFM (rest) have been analysed using analysis of variance—ANOVA with Fisher post-hoc test. Measures of maximum voluntary contraction (peak), endurance from perineometer test and Perfect test parameters (P-power, E-endurance, R-repetitions, F-fast) and also the results of the questionnaires UDI6, ICIQ-SF were analysed using Mann–Whitney test for comparing groups and Wilcoxon test for comparing results before and after 6 weeks of therapy.

## Results

### Description of the patient population participating in the study

The groups were no significantly different in terms of age, height, weigt, BMI at the beginning of the research. The characteristics of the women participating in the study are presented in Table 1.

**Table 1:** Characteristics of women participating in the study.

	Exercise group (n = 30) Mean with SD	Control group (n = 26) Mean with SD
Age [years]	32.21 ± 2.86	30.76 ± 3.82
Height [cm]	166.70 ± 4.5	166.58 ± 4.90
Weight [kg] at the beginning of the study	66.02 ± 12.04	66.19 ± 10.59
BMI [kg/m^2^] at the beginning of the study	23.70 ± 3.85	23.81 ± 3.33
Education level	n/%	n/%
Secondary	0	2/7.69%
Higher	30/100%	24/92.31%

### Perineometer test

The analysis of the maximum voluntary contraction and the endurance of the pelvic floor muscles measured with a perineometer in both groups showed a statistically significant improvement 6 weeks after the first measurement (Tables 2, 3). When comparing the endurance of the pelvic floor muscles between the exercise and control groups, a statistically significant difference is noted, both during the first and second measurement. The exercise group at the beginning of the project had better perineal muscle endurance, and after 6 weeks the difference between the groups was even greater (Tables 2, 3, 4). In the exercise group, the average value of the maximum vaginal squeeze pressure (22.43 cmH_2_O) is higher compared to the non-training group (19.94 cmH_2_O), but the results did not show statistical significance. There were no statistically significant differences between the groups during both measurements in the values of resting pressure of the PFM (Table 4).

**Table 2:** Results of the resting pressure, peak pressure and endurance of the PFM using the perineometer in the exercise group.

Parameter	Exercise group (n = 30)					
	Average	SD	Median	Min	Max	*p* value
**Rest [cmH**_**2**_**0]**						
Before	22.42	5.88	22.80	7.60	36.60	0.716*
After	22.13	4.66	21.35	13.80	32	
**PEAK [cmH**_**2**_**O]**						
Before	18.91	8.69	19.17	5.10	35.37	0.018**
After	22.43	9.89	21.23	6.57	48.87	
**Endurance [s]**						
Before	3.53	1.52	3.45	0.50	8	0.000**
After	6.11	2.62	5.9	1	13.50	

Values statistically significant are in bold.

*Anova with repeated measures with Fisher post-hoc test.

**Wilcoxon test.

**Table 3:** Results of the resting pressure, peak pressure and endurance of the PFM using perineometer in the control group.

Parameter	Control group (n = 26)					
	Average	SD	Median	Min	Max	*p* value
**Rest [cmH**_**2**_**0]**						
Before	22.96	5.34	22.50	12.80	33.40	0.242*
After	24.07	5.14	23.95	15	37.10	
**PEAK [cmH**_**2**_**O]**						
Before	16.96	9.51	16.57	5.73	45.67	0.019**
After	19.94	9.96	19.38	5.73	42.63	
**Endurance [sek.]**						
Before	3.18	2.26	2.60	0.50	11.10	0.000**
After	4.39	3.29	3.45	1.50	15	

Values statistically significant are in bold.

*Anova with repeated measures with Fisher post-hoc test.

**Wilcoxon test.

**Table 4:** Analysis of differences between groups in terms of the results of the resting pressure, peak pressure and endurance of the PFM using the perineometer.

	Exercise group n = 30 Median	Control group n = 26 Median	*p* value
Rest [cmH_2_0] before	22.80	22.50	0.775*
Rest [cmH_2_0] after	21.35	23.95	0.217*
PEA [cmH_2_O] before	19.17	16.57	0.312**
PEA [cmH_2_O] after	21.23	19.38	0.336**
Endurance [s] before	3.45	2.60	0.047**
Endurance [s] after	5.90	3.45	0.001**

Values statistically significant are in bold.

*Anova with repeated measures with Fisher post-hoc test.

**Mann–Whitney test.

### Perfect test

When assessing the pelvic floor muscles in each group using the Perfect test, a statistically significant improvement was noticed in all parameters after 6 weeks (Tables 5, 6). However, a statistically significant difference between the exercise group and the control group is noted only in PFM endurance (Table 7).

**Table 5:** PFM assessment results based on Perfect test in the exercise group.

Parameter	Exercise group (n-30)					
	Average	SD	Median	Min	Max	*p* value
**P-power**						
Before	2.93	0.98	3	1	5	0.001*
After	3.47	0.78	3.5	2	5	
**E-endurance [s]**						
Before	3.42	1.29	3	0.5	6	0.000*
After	5.77	2.11	5	1	10	
**R-repetitions**						
Before	4.23	1.22	4	2	7	0.000*
After	5.63	1.35	6	3	10	
**F-fast contractions**						
Before	8.33	2.35	10	1	10	0.000*
After	9.93	0.37	10	8	10	

Values statistically significant are in bold.

*Wilcoxon test.

**Table 6:** PFM assessment results based on Perfect test in the control group.

Parameter	Control group (26 osób)					
	Average	SD	Median	Min	Max	*p* value
**P-power**						
Before	2.92	0.89	3	2	5	0.017*
After	3.19	0.75	3	2	5	
**E-endurance [s]**						
Before	3.27	1.89	3	1	10	0.000*
After	4.19	2.43	4	2	10	
**R-repetitions**						
Before	4.58	1.63	4.5	2	10	0.009*
After	5.31	1.81	5	2	10	
**F-fast contractions**						
Before	8.11	2.57	10	2	10	0.007*
After	9.39	1.70	10	3	10	

Values statistically significant are in bold.

*Wilcoxon test.

**Table 7:** Analysis of differences between groups in the assessment of PFM based on the Perfect test.

	Exercise group n = 30 Median	Control group n = 26 Median	*p* value
P-power before	3	3	0.856*
P-power after	3.5	3	0.165*
E-endurance before [s]	3	3	0.194*
E-endurance after [s]	5	4	0.001*
R-repetitions before	4	4.5	0.473*
R-repetiotions after	6	5	0.265*
F-fast contractions before	10	10	0.850*
F-fast contractions after	10	10	0.115*

Values statistically significant are in bold.

*Mann–Whitney test.

### Analysis of the results obtained from the UDI 6 and ICIQ-SF questionnaires

Table 8 presents data on the UDI 6 and ICIQ-SF questionnaires. In the exercise group the number of points obtained with the UDI 6 questionnaire ranged from 0–7 points (0–2 points after 6 weeks) and 0–5 points in the control group (0–4 points after 6 weeks). When analyzing the data from the ICIQ-SF questionnaire in the exercise group, the results were between 0–11 points (after 6 weeks, 0–9 points), in the control group 0–12 points (after 6 weeks 0–9 points). In both groups there was a statistically significant difference before and after the 6-week study conducted with the UDI-6 and ICIQ-SF questionnaires, which means that subjective measure of severity of urinary continence and quality of life significantly improved over time. In the exercise group, there is a greater difference between the results of the two trials, which allowed us to conclude that exercises reduce discomfort associated with UI. At the beginning of the project no changes were noticed between the groups, considering the results of the questionnaires. At the end of the study, a statistically significant difference occurred between the groups, as assessed by the ICIQ-SF research tool, which means that personal/subjective frequency, severity of urinary intontinence and quality of life related to UI improved at exercise group.

**Table 8:** Analysis of the UDI 6 and ICIQ-SF questionnaires in both groups.

	Exercise group		Control group		*p* value Exercise group	*p* value Control group	*p* value Exercise versus control	*p* value Execise versus control
	Start of the project	End of the project	Start	End	Start versus end	Start versus end	Start	End
UDI 6 (average)	1.87	0.17	1.27	0.58	0.001*	0.041*	0.396**	0.096**
ICIQ-SF (average)	2.93	0.43	2.38	1.65	0.001*	0.035*	0.538**	0.024**

Values statistically significant are in bold.

*Wilcoxon test.

**Mann–Whitney test.

### Analysis of the results of urinary incontinence in different stages of women's lives

Table 9 summarizes the time of onset and changes in the incidence of urinary incontinence at various times of the life of women participating in the project. The values in parentheses indicate the number of women who experienced urinary leakage for the first time during the corresponding period. The following lines show how many of them had continuous episodes of urine loss. For example: in the exercise group 6 women had urinary incontinence before pregnancy and 5 of them had the problem during pregnancy as well as within 1–6 weeks after delivery and 6–8 weeks after delivery. Only one of them experienced the problem 3 months after delivery. The table shows that participation in exercise 6–8 weeks after vaginal delivery significantly reduces the problem of urine loss 3 months after delivery. In total 56 women from both groups, 32 of them (57.14%) experienced symptoms of urinary incontinence during pregnancy. In 21 women, loss of urine was also repeated in the puerperium—constituting 65.62% of those who experienced incontinence during pregnancy.

**Table 9:** Time of onset and incidence of changes in urinary incontinence in subsequent periods of women's life in both groups.

	Exercise group [n]			Control group [n]		
Before pregnancy	(6)			(5)		
Pregnancy	5	(14)		4	(9)	
1–6 weeks after delivery	5	9	(3)	5	3	(3)
6–8 weeks after delivery	5	6	2	4	3	2
3 months after delivery	1	1	0	4	3	1

## Discussion

The International Continence Society recommends pelvic floor muscle training for women after their first natural delivery to reduce and minimize the risk of developing urinary incontinence. All women after vaginal delivery should participate in a PFM training program, regardless of whether or not they have urine loss^29^.

Scientists have noticed that the strength of the PFM decreases after the first vaginal delivery^29,30^. Many women are not informed about PFM training and thus have a higher risk of developing UI^29^. PFM exercises are recommended as the first line of treatment in pelvic floor dysfunction, incl. UI^31,32^, as well as during pregnancy and after delivery^32^. Therefore, it was decided to conduct the research in order to educate women about PFM using the BeBo Concept as a method which is used to prevent and treat pelvic floor muscle dysfunction.

In our research 65% of primiparous women complaining about UI during pregnancy had episodes of urine loss also in the postpartum period. These data are similar to the results obtained by Leroy et al. They noticed that in 70.1% of women, urinary incontinence appeared during pregnancy and continued in the postpartum period^15^. For this reason, it is important to guide women about PFM.

A significant reduction in both vaginal resting pressure and the strength and endurance of the perineal muscles was reported by comparing the period from mid-pregnancy to 6 weeks after vaginal delivery in the studies of Hilde et al. Continent women showed a stronger pelvic floor compared to their incontinent counterparts^30^. However, there is a thesis in the literature that an increase in strength of PFM does not necessarily correlate with better functioning^33^. PFM strengthening exercises reduce the symptoms of SUI, but many studies have shown that the strength of PFM contraction does not always correlate with the state of continence or the effect on the urethra^3^.

In our study, we noticed progres in subjective (UDI-6, ICIQ-SF) and objective (perineometr, Perfect test) measurement values in both groups. There were no significant differences in PFM strength between groups, yet statistically significant improvement in strength was observed in both groups. These results show that up to 3 months after childbirth, self-regeneration in the pelvic floor muscles is observed. Similar resultes presented Colla et al. Using a perineometer, they observed that the strength of PFM contraction appeared to spontaneously recover 1 to 3 months after delivery. They emphasized that time is the factor that influences the functioning of PFM, which tend to recover physiologically^34^.

In our findings we also observed that urinary incontinence symptoms and impact of UI on the quality of life significantly improved in all patients. However, in the exercise group it was statistically significantly better than in the control group (ICIQ-SF score). Many women who experienced loss of urine 6–8 weeks postpartum (84.66%) and who participated in the BeBo physiotherapy program, experienced recovery after 6 weeks of exercise. Therefore, it should be stated that participation in the exercises had a positive impact on the functioning of PFM and quality of life.

Similar results showed studies by Zarawski et al. They confirm that PFM training is an effective method of preventing urinary incontinence in pregnancy and after childbirth. Regular exercise reduces the frequency and intensity of urine leakage during these two periods. The authors also point out the development of urinary incontinence in women (68.97%) who did not undertake PFM exercises and who suffered perineal rupture or incision during labor^35^.

Also another study by El Nahas et al. support our results. They evaluated PFM exercise in 50 primiparous women with mild stress urinary incontinence 3 months postpartum. Perineometer and ultrasound were used to assess the perineal muscles. There was a statistically significant increase in the strength and thickness of the pelvic floor muscles in group A (with aerobic exercise program, additionally with biofeedback-assisted PFM exercises) and in group B (only undertook Kegel exercises). However, in group A the results were more favorable^36^.

In our study women from the exercise group were doing homework exercise: mobilization of the pelvis, PFM exercises incl. This could have contributed to achieving good results in this group. Likewise, Ahlund et al. indicate that home-based PFM training in primiparous women is effective. The maximum voluntary contraction and endurance of PFM showed a statistically significant increase between 3 and 9 months after delivery. An improvement in symptoms associated with loss of urine (ICIQ FLUTS) was also noted. There was no correlation between maximum voluntary contraction and urinary incontinence reporting. Improvement in the control group (given only written instructions of PFM training) may also be related to the normal reparative process^37^. A similarity can be seen between that paper and the results presented in our study. However, the time and duration of the postpartum measurements performed were different. According to our results, there was also an improvement in the parameters of the pelvic floor muscles in both groups, the control group did not exercise the pelvic floor muscles, which may indicate a progressive regeneration process.

Research shows that increasing body awareness by training the PFM in women can reduce postpartum urinary incontinence^29^. The BeBo Concept used in the study pays attention to, among others, proper body posture and PFM activation. This combination had a positive impact on the quality of life and reduction of incidents of urine leakage in women participating in the exercise group.

Moreover, Kahyaoglu Sut et al. also noted a positive effect of PFM exercises performed during pregnancy and in the postpartum period. In the 6–8 week postpartum period, the strength of PFM was also statistically significantly greater than in the control group^38^.

PFM exercises help women with all types of UI, but they are most beneficial for women with stress urinary incontinence who participate in a supervised perineal muscle training program for at least 3 months^39^. In the presented study, 2 women from the exercise group still declared symptoms of urine loss after participating in a 6-week training course. The period of exercise may have been too short for them and the desired effect could be achieved after longer, more systematic work.

The studies of Junginger et al. show that the contraction of approximately 50% of the maximum strength of PFM causes a significant elevation of the bladder neck in most women and does not affect breathing. In contrast, maximum contraction of the perineal muscles causes the IAP to increase and the bladder neck does not rise^40^. In the presented studies, exactly this kind of PFM contraction (50% maximal PFM contraction) was used in the instructions for home exercises for women in the exercise group.

There is no uniform information in the literature concerning the most advisable number of series and repetitions of PFM exercises. In this study, the main emphasis was placed on prophylaxis, which is why the participants were asked to exercise once a day. Greater effects might be obtained if the program lasted longer than 6 weeks or the number of series was increased. It should be added, however, that apart from exercises focused on the muscles of the pelvic floor, the homework also included exercises that mobilize the pelvis and balance exercises.

Most studies recommend 10 repetitions of PFM training 1–3 times a day in the case of stress urinary incontinence^41^. Following the latest research results women in this study from the exercise group were asked to contract the pelvic floor muscles up to 50%, synchronized with breathing^42^. The strength and endurance of the perineal muscles was built up gradually, due to the fact that some women after childbirth were not able to hold the tension of these muscles for a long time. In the second week they started to hold PFM tension for 3 s and in the 4th week they were asked to hold it for 5 s.

In summary, our project has some limitations, which could be taken into account in future research. Firstly, long-term effects of exercises were not included because this study was finished after 6 weeks intervention. Secondly, the research was not blinded. A blinded study would not be possible to conduct, as one group realized physiotherapy program using BeBo Concept. The exercise group had twice a week individual sessions and homework, whereas the control group did not participate in any research procedures until their follow up visit. This hyperawareness and attentiveness to pelvic floor could have an impact on the results. However, we need to remember that exercise group not only had to activate PFM, but also paid attention to correct body posture and did five groups of exercises according to the Bebo Concept. This method is based on holistic approach to the human body, which we consider to be a significant factor to include in PFM exercises in order to achieve benefits like prevention or treatment of UI and restoration of the quality of life.

## Conclusions

1. In all women after natural childbirth, regardless of treatment, measured parameters improvements were seen, yet they were slightly more explicit in those who participated in the Bebo Concept exercise group.
2. In both groups (exercise and control group) we observed improvement regarding subjective occurence and severity of UI as well as impact of urine leakage on the quality of life. The difference between groups was statistically significant by ICIQ-SF. In the end of the study, in the exercise group more women who experienced loss of urine at the begining improved urinary continence after 6 week program.

Practical conclusions from the project:

1. It can be concluded that exercises according to the BeBo Concept are a method that positively influences the improvement of most of the analyzed parameters, therefore it seems that the exercise program used in the research should be proposed to women after natural childbirth.
2. The conducted research shows that in all maternity wards, women should be informed about the prophylaxis of pelvic floor muscle dysfunction. Education on this topic and teaching appropriate exercises is recommended.
